# Prolonged Ischemia Induces Cellular Stress, Stimulates Extracellular Matrix Remodeling and Compromises the Viability of Human Cancellous Bone Grafts

**DOI:** 10.3390/cells15141261

**Published:** 2026-07-14

**Authors:** Maximilian M. Menger, Tina Histing, Franziska Poeske, Lina P. Schäfer, Konrad Steinestel, Lena-Maria Löwer-Kiem, Michael D. Menger, Matthias W. Laschke, Patrick Münzer, Oliver Borst, Benedikt J. Braun, Sabrina Ehnert, Steven C. Herath

**Affiliations:** 1 Department of Trauma and Reconstructive Surgery, BG Clinic Tuebingen, Eberhard Karls University Tuebingen, 72076 Tuebingen, Germany; 2 Department of Trauma and Reconstructive Surgery, BG Clinic Tuebingen, Siegfried Weller Institute for Trauma Research, Eberhard Karls University Tuebingen, 72076 Tuebingen, Germany; 3 Institute of Pathology and Molecular Pathology, Bundeswehrkrankenhaus Ulm, 89081 Ulm, Germany; 4 Institute for Clinical and Experimental Surgery, Saarland University, PharmaScienceHub (PSH), 66421 Homburg, Germany; 5 DFG Heisenberg Group Cardiovascular Thrombo-Inflammation and Translational Thrombocardiology, University of Tuebingen, 72076 Tuebingen, Germany; 6 Department of Cardiology, Angiology and Cardiovascular Medicine, University Hospital of Tuebingen, 72076 Tuebingen, Germany

**Keywords:** autologous, bone graft, ischemia, viability, cellular stress, extracellular matrix

## Abstract

Fracture healing failure remains a major complication in trauma and orthopedic surgery. The transplantation of autologous cancellous bone grafts represents the gold standard for the treatment of atrophic non-unions. However, during revision surgery the grafts can be exposed to a significant period of intraoperative ischemia, which may have detrimental effects on their quality and functionality. Therefore, we analyzed the effects of different periods of ischemia (0, 30, 60 and 90 min) on cellular stress, gene expression and viability of the bone grafts, to determine a critical ischemia time window for transplantation. Graft samples were harvested from 24 patients undergoing revision surgery due to bone healing failure. Analyses included mRNA profiler arrays, reverse transcription polymerase chain reaction (RT-PCR) and immunohistochemistry. Ischemia lasting 60 min or longer induced the expression of stress-induced genes, such as *JUN* and *DUSP1*. This was associated with early cellular stress within the grafts, as indicated by the presence of hypoxia-inducible factor (HIF)-1α-positive cells and an increased number of senescent p16-positive cells at early time points of ischemia. Additional analyses revealed a significantly higher number of apoptotic cleaved caspase-3-positive cells at 60 and 90 min of ischemia, demonstrating a compromised viability of the grafts. Moreover, RT-PCR analyses revealed a shift from a pro-osteogenic towards a pro-chondrogenic extracellular matrix (ECM) gene expression profile. Taken together, periods of ischemia of 60 min or longer after tissue harvesting should be avoided during cancellous bone graft transplantation to preserve graft viability.

## 1. Introduction

Despite an increasing knowledge of the cellular and molecular mechanisms of bone regeneration, 2–10% of all fractures result in delayed healing or non-union formation [1,2]. Thus, non-unions remain a major burden in trauma and orthopedic surgery. For the patient, failed fracture healing results in substantial pain and an impaired function of the affected limb with a subsequent reduction in the quality of life. Moreover, a prolonged rehabilitation process as well as the loss in work force and productivity cause a significant economic burden to our society and health care system [3,4].

The transplantation of cancellous autologous bone grafts remains the gold standard in the treatment of atrophic non-unions and substantial bone tissue defects. Autologous bone grafts exhibit a high regenerative activity, due to their osteogenic, osteoinductive and osteoconductive properties [5,6]. In fact, bone grafts provide structural support, induce *de novo* bone formation by pro-osteogenic precursor cells, and stimulate the recruitment of mesenchymal stem cells (MSCs). Moreover, the grafts provide a connective tissue matrix serving as a scaffold for hosting cells to induce bone regeneration [6,7,8,9]. Typical donor sites include the iliac crest, the distal femur and the proximal tibia [6].

However, depending on the type of procedure and the available personnel, there may be a significant delay between the harvesting of the cancellous bone graft and its transplantation into the bone defect. A variety of previous studies aimed to improve the viability of autologous bone grafts by modifying the storage conditions, including temperature or storage mediums such as saline, distilled water or glucose solution [10,11,12,13]. However, there is still no consensus on the ideal intraoperative management of autologous bone grafts.

During intraoperative *ex situ* storage at room temperature, the supply of vital nutrients and oxygen is interrupted, resulting in tissue ischemia. This, in turn, may lead to significant tissue damage, impairing the functionality and viability of the graft [14]. Hence, the aim of the present study was to determine the critical duration of the ischemic threshold for the transplantation of autologous bone grafts. Therefore, in the present study we analyzed the effects of different periods of ischemia on cellular stress, gene expression and viability of cancellous bone grafts using mRNA profiler arrays, reverse transcription polymerase chain reaction (RT-PCR) and immunohistochemistry. We hypothesized that prolonged ischemia induces cellular stress and affects the viability of cancellous bone grafts.

## 2. Material and Methods

### 2.1. Ethics

Patients undergoing revision surgery due to delayed bone healing or non-union formation in a level 1 trauma center were included in the present study. The patients’ written informed consent was obtained. The analyses of the patients’ specimens and blood samples from healthy volunteers were carried out in accordance with the corresponding ethics vote (240/2022BO2 and 844/2020BO2, respectively) of the local Ethics Committee of the Eberhard-Karls-University, Tuebingen, Germany.

### 2.2. Graft Retrieval and Experimental Protocol

**Figure 1:**
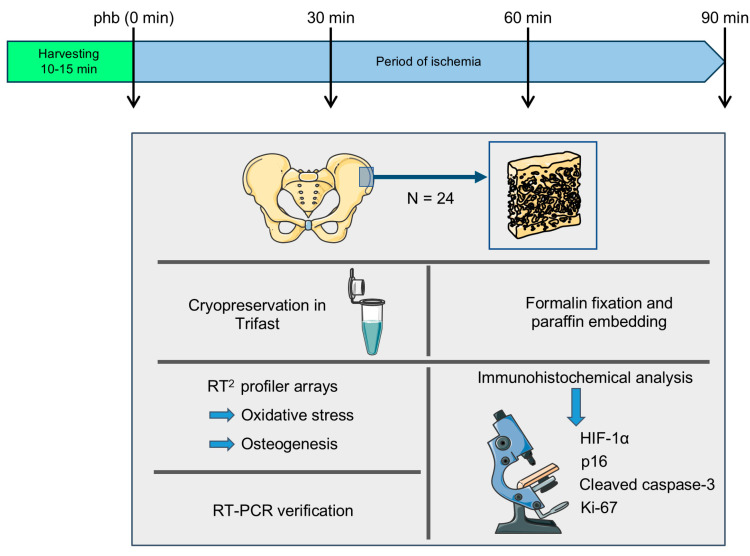
Experimental protocol. Samples of autologous bone grafts were harvested in patients undergoing revision surgery due to compromised fracture healing and non-union formation. The harvesting procedure took approximately 10–15 min. Afterwards the sample were stored for 0 (phb), 30, 60 and 90 min in sterile Ringers’ solution under room temperature. Subsequently, the samples were cryopreserved in Trifast. Profiler arrays for markers of oxidative stress and osteogenesis were performed, with a subsequent RT-PCR verification. Moreover, the samples were analyzed by immunohistochemistry with HIF-1α, cleaved caspase-3, Ki-67 and p16 for the quantification of hypoxia, apoptosis, proliferation and senescence within the bone grafts.

Small tissue samples of autologous bone grafts (1–2 cm^3^) were retrieved from patients undergoing revision surgery due to delayed bone healing or non-union formation (total number of grafts: N = 24). The samples were either obtained from the iliac crest (N = 22) or from long tubular bones (N = 2). The procedure of harvesting the autologous bone grafts took approximately 10 to 15 min. Subsequently, the grafts were stored for 0, 30, 60 and 90 min in sterile Ringer’s solution at room temperature and then processed for further analyses. Since the 0 min group already experienced relevant ischemia during tissue harvesting, we defined this group as post-harvest baseline (phb). These included cryopreservation in Trifast for total mRNA extraction and cDNA synthesis for RT^2^ profiler arrays and RT-PCR, as well as formalin fixation, paraffin embedding, and tissue sectioning for immunohistochemistry (Figure 1). The tissue samples were randomly assigned to the different analysis groups.

### 2.3. RT^2^ Profiler™ PCR Array Human Osteogenesis and Oxidative Stress

For exploratory and screening purposes for identifying pathways and genes of interest, an array analysis was performed. Total mRNA was isolated by phenol-chloroform extraction with following purification using the RNeasy Mini Kit (#74104, Qiagen, Hilden, Germany). Total mRNA content was quantified photometrically. After confirming the mRNA integrity by agarose gel electrophoresis, the RT^2^ First Strand Kit was used to transcribe pooled mRNAs (equal ratios of N = 6 donors) into cDNA (#330404, Qiagen). Uniform cDNA synthesis was confirmed by 18s RT-PCR before performing the RT^2^ Profiler™ PCR arrays Human Nitric Oxide Signaling Pathway (#330231, GeneGlobe-ID PAHS-062ZC, Qiagen) and Human Osteogenesis (#330231, GeneGlobe-ID PAHS-026ZC, Qiagen). For this purpose, 1 µg of the cDNA pool mixed with the RT^2^ SYBR Green ROX qPCR Mastermix (#330523, Qiagen) were applied to each array plate. The sealed plates were run in the StepOnePlus™ Real-Time PCR System (Thermo Fisher Scientific, Waltham, MA, USA) with the protocol provided by Qiagen. After confirming the specificity of the qPCR by melting curve analysis, the obtained data were analyzed using the online tool from GeneGlobe (https://geneglobe.qiagen.com/us/analyze, 26 February 2024). Relative expression changes were used for a gene set enrichment analysis (GSEA—Panther Pathway) using the online platform https://www.webgestalt.org/# (26 February 2024). The heat map, the gene clustering and the chord diagrams were generated with the help of the online platform https://bioinformatics.com.cn/en (26 February 2024).

### 2.4. RT-PCR

The individual mRNAs were converted into cDNA using the first strand cDNA synthesis kit (ThermoFisher Scientific, Waltham, MA, USA) according to the manufacturers’ instructions (N = 10). RT-PCR was carried out in duplicates (n = 2) with the 2x Red Taq Mastermix (Biozym, Oldendorf, Germany). The mean of the duplicates was determined for each patient. Optimized PCR conditions for each primer set are given in Table 1. PCR products were visualized by ethidium bromide (EthBr) following gel electrophoresis on a 2% agarose gel.

### 2.5. Immunohistochemistry

For immunohistochemical analyses of hypoxia-inducible factor (HIF)-1α and cleaved caspase-3 expression, specimens were fixed in paraformaldehyde (N = 8) and embedded in paraffin. Sections were cut and stained with the corresponding primary (HIF-1α: 1:50; Abcam, Cambridge, UK and caspase-3: 1:100; Cell Signaling Technology, Danvers, MA, USA) and corresponding secondary antibodies. For quantification, positive cells were counted automatically by the QuPath software v.0.5.1 in 3–6 high-power fields (400× magnification) [15].

For the immunohistochemical analyses of Ki67 and p16 expression, additional specimens were fixed in paraformaldehyde (N = 6). Subsequently, decalcification was performed in ethylenediaminetetraacetic acid (EDTA) for preservation of the tissue and staining characteristics [16]. After decalcification, the specimens were embedded in paraffin. Immunohistochemistry was done according to standard protocols as previously described [17] on a BenchMark Ultra Plus Autostainer (Roche, Oro Valley, AZ, USA) using antibodies against p16 (E6H4, CINtec, Roche, Oro Valley, AZ, USA) and Ki67 (Mouse anti-Ki-67, Zytomed Systems, Bargteheide, Germany). For the quantification of p16-positive cells, 3 high-power fields (400× magnification, 0.26 mm^2^) were examined and positive osteoblasts and osteocytes were counted. To assess the Ki67-index, the whole slide was examined, and positive osteoblasts and osteocytes were compared to the number of all osteoblasts/osteocytes.

### 2.6. Blood Coagulation Analysis

For the analysis of the effects of cartilage oligomeric matrix protein (COMP) on blood coagulation, blood from human donors (N = 6) was collected in Na-citrate monovettes and centrifuged for 10 min at 2500 *g* to obtain platelet-free plasma. Subsequently, the prothrombin time (PT) and the activated partial thromboplastin time (aPTT) were measured using a Start4 hemostasis analyzer (Diagnostica Stago, Paris, France) according to the manufacturer’s protocol upon a 10 min pretreatment with 5 µg/mL recombinant human COMP (#PDEH100417, Elabscience, Houston, TX, USA) or the corresponding solvent control (phosphate-buffered saline (PBS)).

### 2.7. Statistical Analysis

Results are presented as box plots with median, interquartile range, extrema and individual data points from each patient (N). Statistical analyses were performed using the GraphPad Prism Software version 8 (GraphPad, San Diego, CA, USA) comparing the mean of each patient (N) for RT-PCR, and blood coagulation as well as immunohistochemical analysis. The non-parametric Wilcoxon test was used to compare early time points of ischemia (phb and 30 min) versus late time points of ischemia (60 and 90 min) and the data of the coagulation analysis. Moreover, data were compared by the non-parametric Friedman test for paired data (changes over time for each donor sample) and Dunn’s multiple comparisons post hoc test. A *p*-value below 0.05 was considered statistically significant.

## 3. Results

### 3.1. Patients’ Characteristics

**Figure 2:**
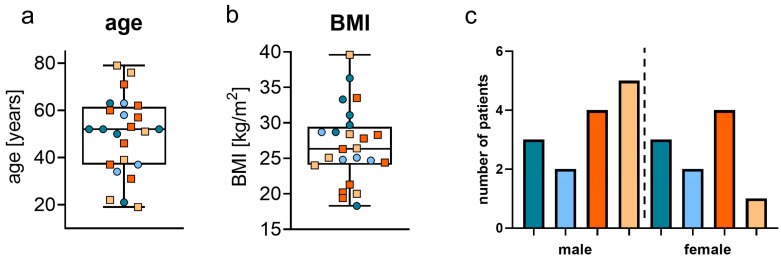
Patients’ characteristics. (**a**,**b**) Age and BMI of the included patients are presented as box plots with individual data points (N = 24). Dark blue circles: samples used for RT-PCR analysis; bright blue circles: samples used for array pooling and additional RT-PCR analysis; dark orange squares: immunohistochemical analysis for HIF-1α and cleaved caspase-3; bright orange squares: immunohistochemical analysis for Ki67 and p16. (**c**) Moreover, the sex of the included patients is illustrated. Dark blue columns: samples used for RT-PCR analysis; bright blue columns: samples used for array pooling and additional RT-PCR analysis; dark orange columns: immunohistochemical analysis for HIF-1α and cleaved caspase-3; bright orange columns: immunohistochemical analysis for Ki67 and p16.

The age of the included patients ranged from 19 to 79 years, with a median age of 52 years. The body mass index (BMI) ranged from 18.3 to 39.6 kg/m^2^, with a median of 26.89 kg/m^2^. Of the 24 patients, 10 patients were female (41.2%) and 14 male (58.8%) (Figure 2a–c). Moreover, the number of patients suffering from common comorbidities was as follows: arterial hypertension (N = 3), type 2 diabetes mellitus (N = 1), chronic kidney failure (N = 1), chronic heart failure (N = 1) and stroke (N = 0).

### 3.2. Profiler Arrays for Oxidative Stress and Osteogenesis

**Figure 3:**
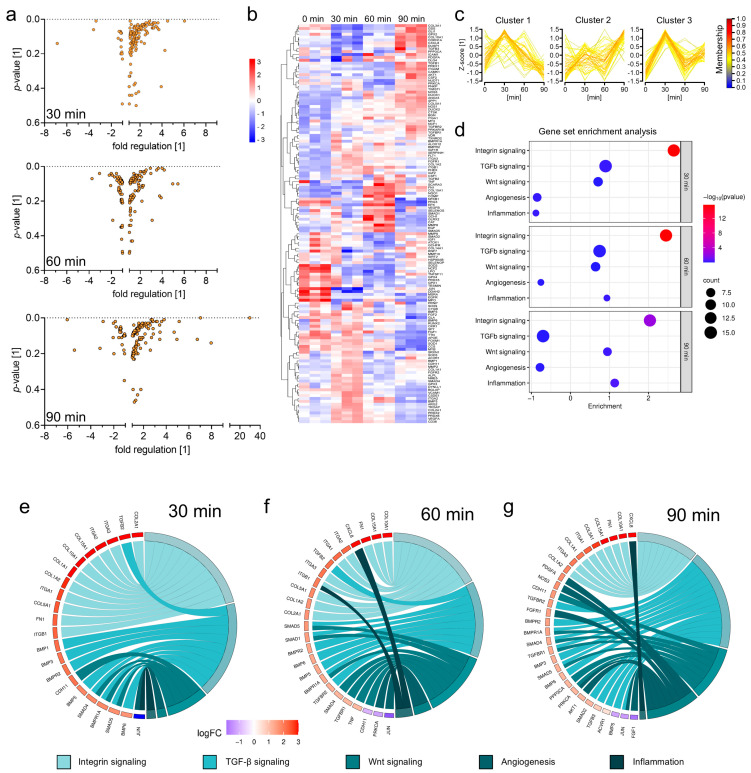
Profiler arrays of oxidative stress and osteogenesis. (**a**) Fold changes in gene expression after 30, 60 and 90 min. (**b**) Heatmap of profiler arrays of oxidative stress and osteogenesis illustrating expression changes. (**c**) Clustering of genes into three main groups according to expression changes. (**d**) Gene Set Enrichment Analysis (GSEA) with the Panther signaling database used as reference: Most regulated genes are involved in integrin, TGF-β, Wnt, angiogenesis, and inflammation signaling via cytokines and chemokines. (**e**–**g**) Expression changes in different genes involving integrin, TGF-β, WNT, angiogenesis and inflammation signaling after 30 min (**e**), 60 min (**f**) and 90 min (**g**) are illustrated.

The RT^2^ profiler arrays revealed distinct changes in gene expression, as early as 30 min after the harvesting of the autologous bone grafts (Figure 3a,b). Genes could be clustered into three main groups based on the expression changes (Figure 3c). A Gene Set Enrichment Analysis (GSEA) with the Panther signaling database used as a reference revealed that most regulated genes are involved in integrin, transforming growth factor (TGF)-β, Wnt, angiogenesis, and inflammation signaling via cytokines and chemokines (Figure 3d). Already at the 30 min time point, alterations affecting integrin signaling and also genes of the extracellular matrix (ECM), e.g., *COL1A1*, *COL1A2*, *COL2A1*, *COL10A1,* or *fibronectin (FN)1*, were strongly upregulated, while genes involved in TGF-β and Wnt signaling were moderately upregulated. In contrast, the effects on gene expression involved in cytokine- and chemokine-mediated inflammation increased over time, with upregulation of *CXCL8* as a key regulator gene that codes for interleukin (IL)-8 transcription (Figure 3e–g).

### 3.3. Inflammation and Cellular Stress Within Cancellous Bone Grafts

**Figure 4:**
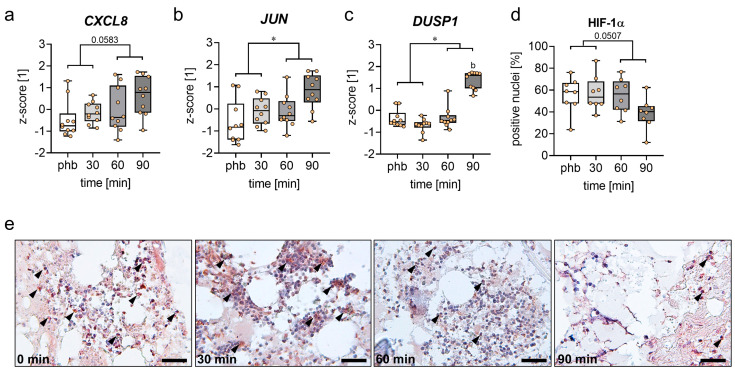
Analysis of inflammation and cellular stress in autologous bone grafts. (**a**–**c**) RT-PCR analyses of *CXCL8* (**a**), *JUN* (**b**) and *DUSP1* (c) gene expression within autologous bone grafts at phb, 30, 60 and 90 min of ischemia. All data were normalized as z-scores and are displayed as box plots with individual data points (N = 10). (**d**) Immunohistochemical analysis of HIF-1α expression within autologous bone grafts at phb, 30, 60 and 90 min of ischemia. All data are displayed as box plots with individual data points (N = 8) (**e**) Representative immunohistochemical images of HIF-1α staining at phb, 30, 60 and 90 min of ischemia. Arrowheads are indicating positive cells. Scale bars: 50 µm. Early (phb and 30 min) and late (60 and 90 min) time points were compared by the non-parametric Wilcoxon test. Individual time points were compared with the non-parametric Friedman test and Dunn’s multiple comparisons post hoc test. * *p* < 0.05: phb and 30 min vs. 60 and 90 min; ^b^ *p* < 0.05 vs. phb and 30 min.

Expression changes in *CXCL8*, *JUN* and *DUSP1* were confirmed by RT-PCR of samples from 10 individual donors. The expression of *CXCL8* showed a strong trend towards upregulation, and the expression of *JUN* and *DUSP1* was significantly upregulated after 60 and 90 min when compared to the early time points phb and 30 min (Figure 4a,b). Notably, the expression of *DUSP1* was most prominently increased after 90 min of ischemia (Figure 4c). Additional immunohistochemical analysis demonstrated that the fraction of HIF-1α-positive cells within the grafts already exceeded 50% at the early ischemic time points of phb and 30 min, indicating cellular stress immediately after removal of the graft (Figure 4d,e).

### 3.4. Viability of Cancellous Bone Grafts

**Figure 5:**
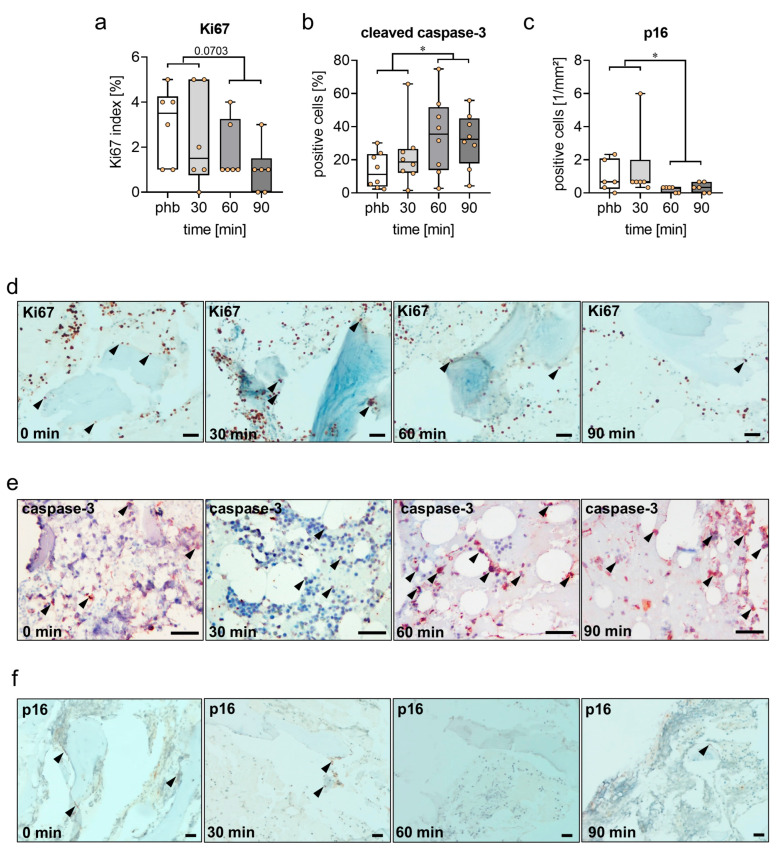
Analysis of the viability of autologous bone grafts. (**a**–**c**) Immunohistochemical analysis of Ki67, cleaved caspase-3 and p16 within autologous bone grafts at phb, 30, 60 and 90 min of ischemia. All data are displayed as box plots with individual data points (N = 6–8). (**d**–**f**) Representative immunohistochemical images of Ki67 (**d**) (scale bars: 20 µm), cleaved caspase-3 (**e**) (scale bars: 50 µm) and p16 (**f**) (scale bars: 20 µm) at phb, 30, 60 and 90 min of ischemia. Arrowheads are indicating positive cells. Early (phb and 30 min) and late (60 and 90 min) time points were compared by the non-parametric Wilcoxon test. Individual time points were compared with the non-parametric Friedman test and Dunn’s multiple comparisons post hoc test. * *p* < 0.05: phb and 30 min vs. 60 and 90 min.

Additional immunohistochemical analyses assessed the viability of the autologous bone grafts. These analyses showed a lower expression of proliferative Ki67-positive cells at the late time points of ischemia when compared to phb and 30 min (Figure 5a,d). Moreover, the number of apoptotic cleaved caspase-3-positive cells was significantly increased at the late time points of ischemia (Figure 5b,e). Furthermore, the number of senescent p16-positive cells was significantly lower at 60 and 90 min after harvesting when compared to the early ischemia time points (Figure 5c,f).

### 3.5. Gene Expression of ECM in Autologous Bone Grafts

**Figure 6:**
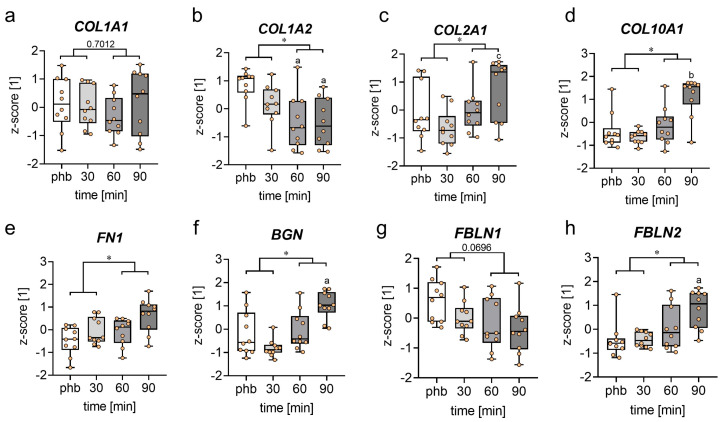
Analysis of ECM gene expression within autologous bone grafts. (**a**–**h**) RT-PCR analyses of *COL1A1* (**a**), *COL1A2* (**b**), *COL2A1* (**c**), *COL10A1* (**d**), *FN1* (**e**), *BGN* (**f**), *FBLN1* (**g**) and *FBLN2* (**h**) gene expression within autologous bone grafts at phb, 30, 60 and 90 min of ischemia. All data were normalized as z-scores and are displayed as box plots with individual data points (N = 10). Early (0 and 30 min) and late (60 and 90 min) time points were compared by the non-parametric Wilcoxon test. Individual time points were compared with the non-parametric Friedman test and Dunn’s multiple comparisons post hoc test. * *p* < 0.05: phb and 30 min vs. 60 and 90 min; ^a^ *p* < 0.05 vs. phb; ^b^ *p* < 0.05 vs. phb and 30 min; ^c^ *p* < 0.05 vs. 30 min.

The analysis of ECM gene expression demonstrated no significant difference in the expression of the pro-osteogenic collagen *COL1A1* with time after harvesting, but a significantly reduced expression of the pro-osteogenic *COL1A2* at the late ischemia time points when compared to the early ischemia time points (Figure 6a,b). In contrast, the expression of the pro-chondrogenic collagens *COL2A1* und *COL10A1* was significantly increased within autologous bone grafts at 60 and 90 min after harvesting (Figure 6c,d). Additionally, the mRNA levels of *FN1*, *fibulin (FBLN)2* and *biglycan* (*BGN)* were significantly elevated at the late time points of ischemia when compared to phb and 30 min (Figure 6e,f,h). In contrast, the expression of *FBLN1* showed a trend towards a decreased with time after harvesting (Figure 6g).

### 3.6. Effects of COMP on Blood Coagulation

**Figure 7:**
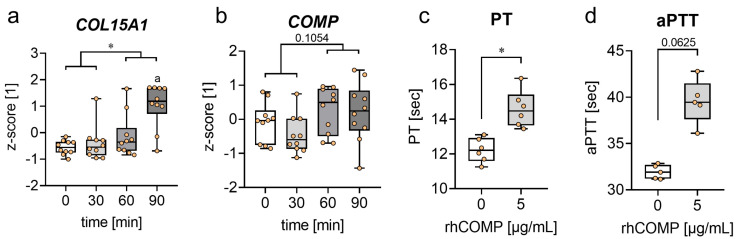
Analysis of *COL15A1* and *COMP* gene expression and coagulation analysis. (**a**,**b**) RT-PCR analyses of *COL15A1* (**a**) and *COMP* (**b**) gene expression within autologous bone grafts at phb, 30, 60 and 90 min of ischemia. All data were normalized as z-scores and are displayed as box plots with individual data points (N = 10). Early (0 and 30 min) and late (60 and 90 min) time points were compared by the non-parametric Wilcoxon test. Individual time points were compared with the non-parametric Friedman test and Dunn’s multiple comparisons post hoc test. * *p* < 0.05: phb and 30 min vs. 60 and 90 min; ^a^ *p* < 0.05 vs. 0 min. (**c**,**d**) PT and aPTT of human plasma with 0 and 5 µg rhCOMP/mL. All data are displayed as box plots with individual data points (N = 5–6). Individual values were compared by the non-parametric Wilcoxon test. * *p* < 0.05: 0 vs. 5 µg rhCOMP/mL.

Additional RT-PCR analyses of markers related to coagulation and angiogenesis revealed a significantly higher expression of *COL15A1* and a trend towards a higher expression of *COMP*, at 60 and 90 min after harvesting, when compared to the early time points of ischemia (Figure 7a,b). The effects of COMP on blood coagulation were further assessed by in vitro coagulation analyses. The results revealed a significantly higher PT and a strong trend towards an increased aPTT in blood clots with COMP treatment when compared to controls (Figure 7c,d).

## 4. Discussion

The present study demonstrates that prolonged ischemia for at least 60 min after bone graft harvesting induces cellular stress, affects ECM gene expression and reduces the viability of cancellous bone grafts. This condition is associated with a significantly upregulated gene expression of *JUN* and *DUSP1*, along with an increased number of apoptotic cells. Moreover, prolonged ischemia decreased the mRNA expression of the pro-osteogenic *COL1A2*, while increasing the expression of the pro-chondrogenic *COL2A1* and *COL10A1*.

Cancellous autologous bone grafts remain the gold standard for the treatment of large bone defects and non-unions, which is due to their excellent osteogenic, osteoinductive, and osteoconductive properties. These characteristics substantially enhance bone regeneration and contribute to overcoming fracture healing failure [18]. Notably, a significant period of intraoperative ischemia can occur between the harvesting of the bone grafts and their transplantation to the recipient site. There is evidence that this ischemic period may compromise the viability of autologous bone grafts and, as a result, the success of the transplantation procedure. In fact, Sun et al. [19] demonstrated in an experimental study in mice that apoptosis and necrosis significantly increases within isolated bone tissue as early as 5 min post-harvesting. Besides this finding, there is no knowledge about the critical threshold for ischemia in human bone tissue as well as on the molecular basis by which ischemia induces damage to the bone graft.

The results of our RT-PCR analysis revealed an upregulation of the pro-inflammatory and cellular stress markers *CXCL8*, *JUN* and *DUSP1* after prolonged ischemia. CXCL8 represents a prototypical chemokine, which belongs to the CXC family. It induces the recruitment and activation of pro-inflammatory cells such as granulocytes and macrophages to the inflammation site. CXCL8 is almost undetectable under physiological conditions, but is rapidly upregulated by pro-inflammatory cytokines such as tumor necrosis factor (TNF)-α and IL-1β [20,21]. CXCL8 exerts its function by interacting with specific cell-surface G-protein-coupled receptors (GPCRs) including C-X-C motif chemokine receptor (CXCR)1 and CXCR2 [22]. Interestingly, CXCL8 expression is upregulated in organ transplantation during both hypoxia and subsequent tissue reperfusion. The infiltration of neutrophilic granulocytes induced by CXCL8 can exert tissue damage by the release of reactive oxidative species (ROS) and destructive enzymes [23]. Accordingly, several preclinical and clinical studies indicate that an upregulation of CXCL8 is associated with critical ischemia and transplantation failure. Klimiec-Moskal et al. [24] demonstrated that high CXCL8 levels within the blood plasma samples of patients suffering from ischemic stroke predict poor functional outcome. Furthermore, the CXCL8 receptor blocker, reparixin, improved the long-term neurological recovery in a cerebral ischemia model in rats [25]. In lung transplant recipients, elevated CXCL8 levels and increased neutrophil counts have been detected in bronchoalveolar lavage (BAL) fluid from patients with bronchiolitis obliterans syndrome (BOS) and restrictive allograft syndrome (RAS), two forms of chronic rejection that represent major causes of long-term mortality after transplantation [23,26].

c-JUN and its related pathway, the c-Jun N-terminal kinase (JNK) pathway, are activated by a variety of stimuli including oxidative stress, heat and osmotic shock as well as ischemia–reperfusion injury of brain and heart tissue [27]. Moreover, there is evidence that JNK activation plays a crucial role in both death receptor-initiated extrinsic and mitochondrial intrinsic pathways that trigger apoptosis [28]. In line with these findings, our immunohistochemical analysis demonstrated a significantly higher number of apoptotic cells after prolonged ischemia. The induction of *CXCL8* and *JUN* was most likely caused by early hypoxia and oxidative stress within the transplants, as indicated by the substantial presence of HIF1-α-positive cells at early ischemic time points. Of note, these observed HIF1-α-positive cells at early time points may be the result of the required time (10–15 min) for bone graft harvesting, which already affects the bone graft viability. Hence, the proposed ischemic threshold of 60 min or longer should be clearly considered as 60 min of storage after the harvesting procedure.

In addition, a significantly higher number of senescent p16-positive cells was observed during early when compared with late time points of ischemia. This observed decline in p16-positive cells with increasing cleaved caspase-3 positivity may reflect a loss of a vulnerable p16-positive cell population during prolonged ischemia. This hypothesis is in line with previous reports, which describe a transition from cellular senescence towards apoptosis [29,30]. Panneer Selvam et al. [29] even suggest that the inhibition of p16 leads to a switch of the cellular phenotype from senescence to apoptosis. This underlines the potential functional link between cellular senescence and apoptosis, including the apoptotic elimination of senescent cells under sustained stress conditions.

Interestingly, our RT-PCR analysis also showed the significant upregulation of *DUSP1* during later stages of ischemia. DUSP1 is expressed during oxidative stress and is responsible for the inhibition of mitogen-activated protein kinases (MAPKs), such as JNK, by dephosphorylation during inflammation [31]. Moreover, DUSP1 has been shown to play a crucial role in the regulation of the inflammatory response of lipopolysaccharide genes, including IL-6 and IL-10 [32]. In the present study the upregulation of DUSP1 after prolonged ischemia indicates a persisting inflammatory state, which may have also contributed to the increased number of apoptotic cells observed after prolonged ischemia.

Additional RT-PCR analyses revealed significant alterations in the expression of ECM-related genes during the time course of ischemia. Collagens are abundant within the extracellular matrix and play a critical role in maintaining the structural integrity of the body. Type I collagen, a triple helical molecule synthesized from the two genes, *COL1A1* and *COL1A2,* is the most common collagen within the human body and represents the major structural protein of bone tissue, comprising 90% of its organic matrix [33]. Notably, it is assembled as a heterotrimer composed of two proα1(I) and one proα2(I) chains, which undergo post-translational modifications such as hydroxylation and glycosylation [34]. Osteogenesis imperfecta, an inherited skeletal dysplasia characterized by bone fragility and skeletal deformities, is caused in most cases by mutations within the *COL1A1* and *COL1A2* genes. Accordingly, it is well established that type I collagen plays a crucial role in bone development and mineralization [35]. Moreover, Besio et al. [36] demonstrated in a transgenic osteogenesis imperfecta mouse model that mutations of *COL1A2* result in a delay in fracture healing. Our analysis showed a significantly reduced expression of *COL1A2* after prolonged ischemia, and a slight reduction in *COL1A1* expression at 90 min of ischemia. On the other hand, the expression of genes of collagens predominant in cartilage tissue, such as *COL2A1* and *COL10A1* [37], were significantly increased at 60 and 90 min of ischemia. These findings indicate a reduced pro-osteogenic capacity of the bone grafts at later stages of ischemia with a shift in ECM-related gene expression toward a pro-chondrogenic and away from a pro-osteogenic profile.

Further changes in ECM-related gene expression included a significant upregulation of *FN1* and *BGN* after prolonged ischemia. Notably, FN1 plays not only a vital role in cellular adhesion and growth [38], but is also upregulated during myocardial ischemia and ischemia–reperfusion injury due to hypoxic cell stress [39]. In this way, FN1 induces the proliferation of progenitor cells and the migration of inflammatory cells [39,40]. BGN is an ECM-derived danger associated molecular pattern (DAMP), which is proteolytically released during ischemia–reperfusion injury of the kidney and heart failure in myocardial tissue [41,42]. Upon its release, BGN coordinates the inflammatory response as a high-affinity ligand of TLR2 and TLR4 in macrophages [43], regulating the production of various cytokines and immune cell recruitment [41]. Hence, the increased expression of *FN1* and *BGN* within bone grafts during later stages of ischemia does not only reflect hypoxic cellular stress but may also contribute to the stimulation of an inflammatory response within the grafts.

Additional analyses demonstrated a decreased expression of *FBLN1* at later stages of ischemia, whereas the expression of *FBLN2* was significantly increased. Interestingly, previous studies could demonstrate that FBLN1 is directly involved in the process of osteogenesis. Cooley et al. [44] reported that the skulls of FBLN1-deficient mice suffer from a reduced formation of both endochondral and membranous bone tissue. This was most likely due to a reduced BMP-2-mediated induction of osterix, resulting in a compromised osteoblast differentiation [44]. These findings are supported by various in vitro studies highlighting the crucial role of FBLN1 for the pro-osteogenic differentiation of MSCs. FBLN2, on the other hand, is upregulated in various organs, such as the heart, liver, and brain, where it promotes tissue fibrosis and hinders remyelination [45,46,47]. The present data indicate that FBLN2 is also involved in ischemia-induced processes within bone tissue, possibly contributing to fibrotic tissue remodeling under ischemic conditions.

Interestingly, our analysis also demonstrated a significantly increased expression of *COL15A1* within bone grafts exposed to prolonged ischemia. There is evidence that collagen-derived endostatins from collagens 15 and 18 inhibit endothelial cell migration and angiogenesis. Sasaki et al. [48] demonstrated in vitro that endostatins from collagens 15 and 18 inhibit angiogenesis induced by fibroblast growth factor (FGF)-2 or vascular endothelial growth factor (VEGF) in a chorioallantoic membrane model. Adequate angiogenesis and vascularization are crucial for the survival of bone grafts and the subsequent process of bone regeneration [49]. Hence, the increased expression of *COL15A1* during prolonged ischemia may not only impair the pro-angiogenic capacity of the grafts, but also negatively affect graft integration and healing outcome.

Notably, the array data demonstrate an upregulation of ECM-markers as early as 30 min after harvesting. Besides representing an early ischemia-driven response, these results may also reflect an acute stress response triggered by the surgical manipulation. In fact, previous studies demonstrated that mechanical stress induces an alteration in gene expression in a variety of tissues and cells, including the skin, cartilage as well as osteoblasts [50,51,52]. Thus, it cannot be excluded that the early gene expression changes detected in the array analysis as well as the alterations in the tissue viability and gene expression in the RT-PCR analysis are in part due to mechanical injury and ex vivo handling during bone graft harvesting.

Since autologous bone grafts are often transplanted as smaller fragments, hematoma formation and its subsequent coagulation are crucial to keep the grafts in place within the defect site and avoid secondary dislocation. The glycoprotein COMP is not only expressed within cartilage tissue but also appears to play an essential role in the blood coagulation process. In fact, Liang et al. [53] demonstrated that COMP deficiency in mice shortens tail-bleeding and clotting time. Moreover, the authors revealed that a high concentration of exogenously purified COMP increases the PT and aPTT of platelet-free plasma from wildtype mice and humans. Apparently, COMP acts as an endogenous inhibitor of thrombin and suppresses hemostasis [53]. Interestingly, our coagulation analysis confirmed these findings using platelet-free plasma from healthy human donors, with a significantly increased PT and tendency towards a higher aPTT after the application of rhCOMP in vitro. Furthermore, our RT-PCR analysis showed a trend towards a higher expression of *COMP* at later stages of ischemia. It may be speculated that an increased expression of COMP could hamper blood coagulation and may therefore compromise graft stabilization at the transplantation site. However, further experiments and analysis are warranted in the future, such as the measurement of COMP in graft-conditioned medium or tissue lysates with subsequent coagulation analysis to definitely link an increased COMP expression within bone grafts to an impaired blood coagulation.

Several limitations of the present study should be acknowledged. Due to the limited sample size—which was primarily determined by tissue availability—potential confounding effects of patient-specific factors such as age, sex, BMI, comorbidities, medication and donor site could not be evaluated in detail and may have contributed to biological variability. Future studies with larger and more stratified cohorts are needed to analyze the impact of these factors and to further elucidate the understanding of ischemia-induced changes in autologous bone grafts. Moreover, it should be considered that the observed molecular and cellular changes after 60 min may not exclusively reflect tissue ischemia but rather a combination of ischemia-related biological responses (such as hypoxia-driven signaling and metabolic stress) and additional stress induced by ex vivo handling and storage conditions.

## 5. Conclusions

Taken together, the present study demonstrates that prolonged ischemia in bone grafts for at least 60 min after bone graft harvesting stimulates the cellular stress response, reduces graft viability and induces a shift from a pro-osteogenic towards a pro-chondrogenic ECM gene expression profile. Moreover, prolonged ischemia may compromise angiogenesis and hematoma coagulation at the transplantation site. Therefore, revision surgeries involving autologous bone graft transplantation should be carefully coordinated to minimize the ischemia time. Optimizing surgical workflow and graft handling procedures may help to avoid ischemic periods of 60 min or longer, thereby preserving bone graft viability and regenerative capacity. Future studies should also evaluate the effects of ischemia on the pro-osteogenic capacity of autologous bone grafts as well as potential preservation strategies such as hypothermic storage and specialized storage solutions to mitigate ischemia-induced tissue damage.

## Figures and Tables

**Table 1:** List of primers, their sequences, and the corresponding PCR conditions. Primers were designed with the help of primer blast with the respective GenBank accession number listed in the table.

Target	GenBank Accession Number	Sequence Forward Primer	Sequence Reverse Primer	Ta [°C]	# of Cycles	Amplicon Size [bp]
18S	NR_003286	GGACAGGATTGACAGATTGAT	AGTCTCGTTCGTTATCGGAAT	56	25	111
CXCL8 (Interleukin-8)	NM_000584.3	TAGCAAAATTGAGGCCAAGG	AAACCAAGGCACAGTGGAAC	60	40	227
DUSP1	NM_004417.4	CTGTCAACGTGCGCTTCAG	GAAAACGCTTCGTATCCTCCTTTG	63	40	258
JUN	NM_002228.4	GTGCCGAAAAAGGAAGCTGG	CTGCGTTAGCATGAGTTGGC	63	35	175
COL1A1	NM_000088.3	CAGCCGCTTCACCTACAGC	TTTTGTATTCAATCACTGTCTTGCC	56	40	83
COL1A2	NM_000089.4	GGCCCTCAAGGTTTCCAAGG	CACCCTGTGGTCCAACAACTC	60	35	166
COL2A1	NM_001844.4	TGGATGCCACACTCAAGTCC	GCTGCTCCACCAGTTCTTCT	63	40	254
COL10A1	NM_000493.3	AAACCTGGACAACAGGGACC	CGACCAGGAGCACCATATCC	63	40	125
BGN	NM_001711.5	CGCCTCGTGTCTCTGCTGGC	TGGCTGAAGGAACAGCTGAG	58	40	194
FBLN1	NM_006487.3	GAACGCGCTGTGTTGATGTG	ATCCTGCTGATGCCGTCAAA	63	40	136
FBLN2	NM_001004019.2	GCCCCGCGGGTCTTAC	CGCCTCCTCAATGCAGTTCT	63	40	193
COMP	NM_000095.3	CCCAAGTGGGCTACATCAGG	GGTGTCATTGCAGCGGTAA	60	35	164
COL15A1	NM_001855.5	CGCCGCCTTTGTTCCCTG	TGTTCCTCCTTGGTGCCATC	60	40	147

## Data Availability

The original contributions presented in this study are included in the article. Further inquiries can be directed to the corresponding author.

## References

[1] Einhorn T.A., Gerstenfeld L.C. (2015). Fracture healing: Mechanisms and interventions. Nat. Rev. Rheumatol..

[2] Mills L.A., Aitken S.A., Simpson A. (2017). The risk of non-union per fracture: Current myths and revised figures from a population of over 4 million adults. Acta Orthop..

[3] Victoria G., Petrisor B., Drew B., Dick D. (2009). Bone stimulation for fracture healing: What’s all the fuss?. Indian J. Orthop..

[4] Hak D.J., Fitzpatrick D., Bishop J.A., Marsh J.L., Tilp S., Schnettler R., Simpson H., Alt V. (2014). Delayed union and nonunions: Epidemiology, clinical issues, and financial aspects. Injury.

[5] Azi M.L., Aprato A., Santi I., Kfuri M., Masse A., Joeris A. (2016). Autologous bone graft in the treatment of post-traumatic bone defects: A systematic review and meta-analysis. BMC Musculoskelet. Disord..

[6] Schmidt A.H. (2021). Autologous bone graft: Is it still the gold standard?. Injury.

[7] Fillingham Y., Jacobs J. (2016). Bone grafts and their substitutes. Bone Jt. J..

[8] Khan S.N., Cammisa F.P., Sandhu H.S., Diwan A.D., Girardi F.P., Lane J.M. (2005). The biology of bone grafting. J. Am. Acad. Orthop. Surg..

[9] Kamal M., Gremse F., Rosenhain S., Bartella A.K., Holzle F., Kessler P., Lethaus B. (2018). Comparison of Bone Grafts From Various Donor Sites in Human Bone Specimens. J. Craniofacial Surg..

[10] Hassanein A.H., Greene A.K., Arany P.R., Padwa B.L. (2012). Intraoperative cooling of iliac bone graft: An experimental evaluation of cell viability. J. Oral Maxillofac. Surg..

[11] Steiner M., Ramp W.K. (1988). Short-term storage of freshly harvested bone. J. Oral Maxillofac. Surg..

[12] Maus U., Andereya S., Gravius S., Siebert C.H., Schippmann T., Ohnsorge J.A., Niedhart C. (2008). How to store autologous bone graft perioperatively: An in vitro study. Arch. Orthop. Trauma Surg..

[13] Kantor A.H., Uffmann W., Marchand L.S., Haller J.M., Higgins T.F., Rothberg D.L. (2022). What Happens on the Back Table? Viability and Osteogenic Potential of Reamed Autogenous Bone Graft as a Function of Time and Temperature-A Pilot Study. J. Orthop. Trauma.

[14] Eltzschig H.K., Eckle T. (2011). Ischemia and reperfusion--from mechanism to translation. Nat. Med..

[15] Bankhead P., Loughrey M.B., Fernandez J.A., Dombrowski Y., McArt D.G., Dunne P.D., McQuaid S., Gray R.T., Murray L.J., Coleman H.G. (2017). QuPath: Open source software for digital pathology image analysis. Sci. Rep..

[16] Choube A., Astekar M., Choube A., Sapra G., Agarwal A., Rana A. (2018). Comparison of decalcifying agents and techniques for human dental tissues. Biotech. Histochem..

[17] Steinestel K., Bruderlein S., Steinestel J., Markl B., Schwerer M.J., Arndt A., Kraft K., Propper C., Moller P. (2012). Expression of Abelson interactor 1 (Abi1) correlates with inflammation, KRAS mutation and adenomatous change during colonic carcinogenesis. PLoS ONE.

[18] Pape H.C., Evans A., Kobbe P. (2010). Autologous bone graft: Properties and techniques. J. Orthop. Trauma.

[19] Sun Q., Li Z., Liu B., Yuan X., Guo S., Helms J.A. (2019). Improving intraoperative storage conditions for autologous bone grafts: An experimental investigation in mice. J. Tissue Eng. Regen. Med..

[20] Liu Q., Li A., Tian Y., Wu J.D., Liu Y., Li T., Chen Y., Han X., Wu K. (2016). The CXCL8-CXCR1/2 pathways in cancer. Cytokine Growth Factor Rev..

[21] Hoffmann E., Dittrich-Breiholz O., Holtmann H., Kracht M. (2002). Multiple control of interleukin-8 gene expression. J. Leukoc. Biol..

[22] Brat D.J., Bellail A.C., Van Meir E.G. (2005). The role of interleukin-8 and its receptors in gliomagenesis and tumoral angiogenesis. Neuro-Oncology.

[23] Cambier S., Gouwy M., Proost P. (2023). The chemokines CXCL8 and CXCL12: Molecular and functional properties, role in disease and efforts towards pharmacological intervention. Cell. Mol. Immunol..

[24] Klimiec-Moskal E., Koceniak P., Weglarczyk K., Slowik A., Siedlar M., Dziedzic T. (2025). Circulating Chemokines and Short- and Long-Term Outcomes After Ischemic Stroke. Mol. Neurobiol..

[25] Villa P., Triulzi S., Cavalieri B., Di Bitondo R., Bertini R., Barbera S., Bigini P., Mennini T., Gelosa P., Tremoli E. (2007). The interleukin-8 (IL-8/CXCL8) receptor inhibitor reparixin improves neurological deficits and reduces long-term inflammation in permanent and transient cerebral ischemia in rats. Mol. Med..

[26] Verleden S.E., Ruttens D., Vos R., Vandermeulen E., Moelants E., Mortier A., Van Raemdonck D.E., Proost P., Schols D., Verleden G.M. (2015). Differential cytokine, chemokine and growth factor expression in phenotypes of chronic lung allograft dysfunction. Transplantation.

[27] Shvedova M., Anfinogenova Y., Atochina-Vasserman E.N., Schepetkin I.A., Atochin D.N. (2018). c-Jun N-Terminal Kinases (JNKs) in Myocardial and Cerebral Ischemia/Reperfusion Injury. Front. Pharmacol..

[28] Dhanasekaran D.N., Reddy E.P. (2008). JNK signaling in apoptosis. Oncogene.

[29] Panneer Selvam S., Roth B.M., Nganga R., Kim J., Cooley M.A., Helke K., Smith C.D., Ogretmen B. (2018). Balance between senescence and apoptosis is regulated by telomere damage-induced association between p16 and caspase-3. J. Biol. Chem..

[30] Wang D., Liu Y., Zhang R., Zhang F., Sui W., Chen L., Zheng R., Chen X., Wen F., Ouyang H.W. (2016). Apoptotic transition of senescent cells accompanied with mitochondrial hyper-function. Oncotarget.

[31] Liu Y.X., Wang J., Guo J., Wu J., Lieberman H.B., Yin Y. (2008). DUSP1 is controlled by p53 during the cellular response to oxidative stress. Mol. Cancer Res..

[32] Hammer M., Mages J., Dietrich H., Servatius A., Howells N., Cato A.C., Lang R. (2006). Dual specificity phosphatase 1 (DUSP1) regulates a subset of LPS-induced genes and protects mice from lethal endotoxin shock. J. Exp. Med..

[33] Selvaraj V., Sekaran S., Dhanasekaran A., Warrier S. (2024). Type 1 collagen: Synthesis, structure and key functions in bone mineralization. Differentiation.

[34] Marini J.C., Blissett A.R. (2013). New genes in bone development: What’s new in osteogenesis imperfecta. J. Clin. Endocrinol. Metab..

[35] Marom R., Rabenhorst B.M., Morello R. (2020). Osteogenesis imperfecta: An update on clinical features and therapies. Eur. J. Endocrinol..

[36] Besio R., Maruelli S., Battaglia S., Leoni L., Villani S., Layrolle P., Rossi A., Trichet V., Forlino A. (2018). Early Fracture Healing is Delayed in the *Col1a2*^+/*G610C*^ Osteogenesis Imperfecta Murine Model. Calcif. Tissue Int..

[37] Gelse K., Poschl E., Aigner T. (2003). Collagens--structure, function, and biosynthesis. Adv. Drug Deliv. Rev..

[38] Wang Y., Ni H. (2016). Fibronectin maintains the balance between hemostasis and thrombosis. Cell. Mol. Life Sci..

[39] Konstandin M.H., Toko H., Gastelum G.M., Quijada P., De La Torre A., Quintana M., Collins B., Din S., Avitabile D., Volkers M. (2013). Fibronectin is essential for reparative cardiac progenitor cell response after myocardial infarction. Circ. Res..

[40] Chang S.S., Cheng C.C., Chen Y.R., Chen F.W., Cheng Y.M., Wang J.M. (2024). Epithelial CEBPD activates fibronectin and enhances macrophage adhesion in renal ischemia-reperfusion injury. Cell Death Discov..

[41] Poluzzi C., Nastase M.V., Zeng-Brouwers J., Roedig H., Hsieh L.T., Michaelis J.B., Buhl E.M., Rezende F., Manavski Y., Bleich A. (2019). Biglycan evokes autophagy in macrophages via a novel CD_44_/Toll-like receptor 4 signaling axis in ischemia/reperfusion injury. Kidney Int..

[42] Ahmed M.S., Oie E., Vinge L.E., Yndestad A., Andersen G.G., Andersson Y., Attramadal T., Attramadal H. (2003). Induction of myocardial biglycan in heart failure in rats--an extracellular matrix component targeted by AT_1_ receptor antagonism. Cardiovasc. Res..

[43] Schaefer L., Babelova A., Kiss E., Hausser H.J., Baliova M., Krzyzankova M., Marsche G., Young M.F., Mihalik D., Gotte M. (2005). The matrix component biglycan is proinflammatory and signals through Toll-like receptors 4 and 2 in macrophages. J. Clin. Investig..

[44] Cooley M.A., Harikrishnan K., Oppel J.A., Miler S.F., Barth J.L., Haycraft C.J., Reddy S.V., Scott Argraves W. (2014). Fibulin-1 is required for bone formation and Bmp-2-mediated induction of Osterix. Bone.

[45] Khan S.A., Dong H., Joyce J., Sasaki T., Chu M.L., Tsuda T. (2016). Fibulin-2 is essential for angiotensin II-induced myocardial fibrosis mediated by transforming growth factor (TGF)-beta. Lab. Investig..

[46] Ghorbani S., Li C., Lozinski B.M., Moezzi D., D’Mello C., Dong Y., Visser F., Li H., Silva C., Khakpour M. (2024). Fibulin-2 is an extracellular matrix inhibitor of oligodendrocytes relevant to multiple sclerosis. J. Clin. Investig..

[47] Balog S., Fujiwara R., Pan S.Q., El-Baradie K.B., Choi H.Y., Sinha S., Yang Q., Asahina K., Chen Y., Li M. (2023). Emergence of highly profibrotic and proinflammatory Lrat^+^Fbln2^+^ HSC subpopulation in alcoholic hepatitis. Hepatology.

[48] Sasaki T., Larsson H., Tisi D., Claesson-Welsh L., Hohenester E., Timpl R. (2000). Endostatins derived from collagens XV and XVIII differ in structural and binding properties, tissue distribution and anti-angiogenic activity. J. Mol. Biol..

[49] Menger M.M., Laschke M.W., Orth M., Pohlemann T., Menger M.D., Histing T. (2021). Vascularization Strategies in the Prevention of Nonunion Formation. Tissue Eng. Part B Rev..

[50] Ledwon J.K., Kelsey L.J., Vaca E.E., Gosain A.K. (2020). Transcriptomic analysis reveals dynamic molecular changes in skin induced by mechanical forces secondary to tissue expansion. Sci. Rep..

[51] Ott C.E., Bauer S., Manke T., Ahrens S., Rodelsperger C., Grunhagen J., Kornak U., Duda G., Mundlos S., Robinson P.N. (2009). Promiscuous and depolarization-induced immediate-early response genes are induced by mechanical strain of osteoblasts. J. Bone Miner. Res..

[52] Fitzgerald J.B., Jin M., Dean D., Wood D.J., Zheng M.H., Grodzinsky A.J. (2004). Mechanical compression of cartilage explants induces multiple time-dependent gene expression patterns and involves intracellular calcium and cyclic AMP. J. Biol. Chem..

[53] Liang Y., Fu Y., Qi R., Wang M., Yang N., He L., Yu F., Zhang J., Yun C.H., Wang X. (2015). Cartilage oligomeric matrix protein is a natural inhibitor of thrombin. Blood.

